# Prognostic Implications of Pneumothorax and Pneumomediastinum in COVID-19 Pneumonia: A Cross-Sectional Analysis

**DOI:** 10.2174/0118743064418513251104054134

**Published:** 2026-02-02

**Authors:** Dany Gaspard, Nadine Yared, Reem Wehbe, Elias Horanieh, Tarek Bou Dargham, Mohamad Bahij Moumneh, Christine Atallah, Mohammad Fawaz

**Affiliations:** 1 Gilbert and Rose-Marie Chagoury School of Medicine, Lebanese American University, Beirut, Lebanon; 2 Faculty of Medicine and Medical Sciences, University of Balamand, Beirut, Lebanon

**Keywords:** COVID-19, Pneumothorax, Pneumomediastinum, Computed tomography, Respiratory distress syndrome, Reverse transcription polymerase chain reaction, Lung injury, Body mass index

## Abstract

**Introduction:**

The prognostic implications of developing pneumothorax (PT) or pneumomediastinum (PM) in COVID-19 pneumonia remain a topic of debate, with current literature showing conflicting data. We aimed to assess mortality rates and the characteristics of patients with COVID-19 pneumonia who developed these complications compared to those who did not.

**Methods:**

We analyzed data and outcomes for patients aged 18 years or older who were admitted for COVID-19 pneumonia to a tertiary care referral center in Lebanon.

**Results:**

A total of 527 patients (356 men and 171 women) were identified. Events were reported in 43 patients (18 PM, 10 PT, and 15 both). Overall mortality was 28.3%. Mortality was significantly higher in patients with events compared to those without events (69.7% *vs*. 24.5%). Most events occurred in patients with severe lung involvement on computed tomography (CT). Only three patients died within the first 48 hours after the development of an event. Incidence was higher in patients who were overweight or obese and increased with age. Distribution was similar between both genders. Ventilation data showed that 79% of events occurred during non-invasive or invasive mechanical ventilation.

**Discussion:**

Barotrauma events, including PT and PM in COVID-19 pneumonia, were associated with significantly higher mortality and appear to reflect more severe lung involvement. Mortality was not directly caused by the events themselves. A significant proportion occurred in patients on supplemental oxygen or high-flow nasal cannula (21%), highlighting the need for a high index of suspicion for such events even in non-ventilated patients.

**Conclusion:**

There is a strong association between the development of PT and/or PM and mortality in COVID-19 pneumonia.

## 1 INTRODUCTION

Coronavirus disease 2019 (COVID-19), caused by the novel Severe Acute Respiratory Syndrome Coronavirus 2 (SARS-CoV-2), was first identified in December 2019 in Wuhan, China, following a zoonotic transfer from bats to humans [1]. The infection rapidly escalated into a global pandemic as the number of cases increased worldwide [2]. To date, there have been over 7 million deaths [3], with daily fatalities still being reported. Mortality is primarily driven by lung involvement, leading to severe acute respiratory distress syndrome (ARDS) [4]. Radiological manifestations on computed tomography (CT) typically show ground-glass opacities of varying severity [5]. One of the major complications of COVID-19 pneumonia is the development of pneumothorax (PT) and pneumo-mediastinum (PM). Notably, patients with COVID-19 pneumonia have been reported to develop PT or PM even without mechanical ventilation. Recent studies indicate an overall incidence of 1–4%, with some reports showing rates as high as 15% in severe cases [6].

The prognostic implications of developing these two events remain a subject of debate, as studies to date have yielded conflicting results. Three systematic reviews have been published regarding these complications in patients hospitalized with COVID-19 [6-8], with two reporting higher mortality and the third showing no significant difference. All reviews were limited by study design and heterogeneity, and each concluded that further research is needed to better understand this issue. Accordingly, we aimed to investigate the overall mortality of patients who developed these events compared to those who did not and to provide comparative data on this patient population at a tertiary care center in Lebanon.

## 2 METHODS

### 2.1 Study Population

Records from Mount Lebanon University Medical Center, a tertiary care referral center in Hazmieh, Lebanon, were reviewed between September 2020 and May 2021. These dates were chosen because, during this period, the hospital had a dedicated COVID-19 unit, with all patients admitted for COVID-19-related illnesses. This resulted in a high number of admissions and recorded events, allowing for greater validity of the results. Records of all patients admitted with COVID-19 pneumonia were analyzed. The study was designed as a cross-sectional analysis. All adult patients aged 18 years or older with COVID-19 infection confirmed by reverse transcription polymerase chain reaction (RT-PCR) were included, while patients with suspected but unconfirmed COVID-19 infection were excluded. Data collected from each patient included age, sex, body mass index (BMI), smoking status, presence of comorbidities (including diabetes mellitus [DM], hypertension [HTN], chronic kidney disease [CKD], chronic obstructive pulmonary disease [COPD], congestive heart failure [CHF], coronary artery disease [CAD], and malignancy), date of admission, development of pneumothorax (PT), pneumomediastinum (PM), or both, ventilatory status at the time of the event, time between event occurrence and death, severity of lung involvement on chest CT, and final patient disposition (discharge or death).

All patients were treated according to international guidelines during this period, including steroids, tocilizumab in some cases, and supportive care and treatment of other infections or comorbidities. The ARDS-net protocol was used in patients who required mechanical ventilation.

In all patients who underwent a CT of the chest, severity of lung involvement was determined by specialized radiologists using the following arbitrary scale: 0-10% lung involvement: minimal, 11-25% lung involve-ment: moderate, 26-50% lung involvement: important, 51-75% lung involvement: severe, and 76-100% lung involvement: critical.

### 2.2 Statistical Analysis

Statistical analysis was performed using the Statistical Package for the Social Sciences (SPSS) version 25 (SPSS Inc., Chicago, IL, United States). Categorical variables were described as counts with their respective percentages; subsequently, they were analyzed using the χ^2^ test or Fisher’s exact test. Similarly, the percentage comparison between groups was done using the χ^2^ test for association for independent samples. The significance level used for all statistical analyses was 5%.

## 3 RESULTS

A total of 527 patients (356 men and 171 women) were admitted to our institution between September 2020 and May 2021. Of the study population, 43 patients (8.1%) developed an event during their hospital stay. Overall mortality was 28.3%. Patients with events had significantly higher mortality rates compared to controls (30 of 43: 69.7% *vs*. 119 of 484: 24.5%, *p*-value <0.0001). Breakdown of events showed death in 14 of 18 patients with PM (77.7%), 4 of 10 patients with PT (40%), and 12 of 15 patients with PM and PT (80%). The time between the development of an event and death ranged from 1-43 days. One patient died within 24 hours of the development of an event, and two others died within 48 hours after an event. The incidence of events was similar between genders (32 of 356 (9%) in men and 11 of 171 (6.4%) in women (*p*-value > 0.05).

In the cohort, 28.5% of the patients had DM, 54.3% had HTN, 3.8% had CKD, 3.6% had COPD, 6.3% had heart failure, 18.6% had CAD, and 6.6% had malignancy. The distribution of comorbidities was similar between the two groups. There was no statistically significant correlation between the development of an event and any of the comorbidities (Table **1**).

Overall, 29.2% of patients in the cohort were current or previous smokers, 68% had never smoked, while 2.7% of entries were missing this information. There was no significant difference in the occurrence of an event between smokers and non-smokers (7.1% *vs*. 8.9%, respectively, *p*-value >0.05).

**Fig. (1):**
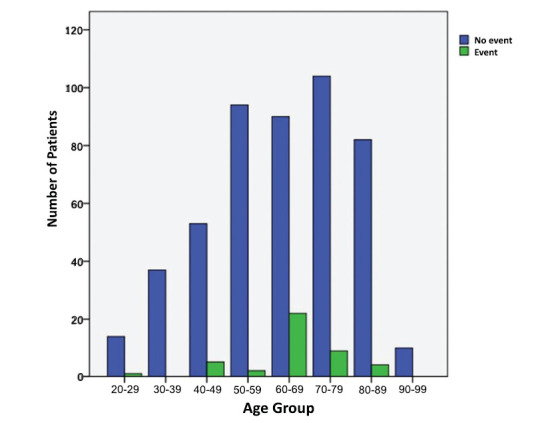
Age groups and distribution of events.

Most events occurred in patients in the 4^th^ to 8^th^ decade of life and increased with increasing age (positive correlation of events with age – *p* < 0.0001), with the highest frequency recorded in the 60-69 age group (19.6%) (Fig. **1**).

Events were more common in patients who were overweight or obese. They were observed in 5.3% of patients with a normal BMI, 11% of patients with a BMI between 25 and 30, 11.7% of patients with a BMI between 30 and 40, and 12.5% of patients with a BMI of more than 40.

CT data were available for 420 patients (73.4%). The distribution of events according to the severity of lung involvement on CT was as follows: 11 of 76 patients (14.5%) with critical involvement, 25 of 172 patients (14.5%) with severe involvement, 1 of 68 patients (1.5%) with important involvement, 3 of 68 patients (4.5%) with moderate involvement, and 1 of 36 patients (2.8%) with minimal involvement. The increased frequency of events in patients with critical and severe involvement was statistically significant (*p* =0.001). The percentage of patients with critical or severe involvement was higher in the population with events (26.8% and 60.9%, respectively) than in the population without events (17.1% and 38.7%, respectively).

Review of ventilation data at the time of occurrence of events in patients with PM showed that 6 were on non-invasive ventilation (NIV), 5 on mechanical ventilation (MV), 6 on non-rebreather face mask (NRFM), and 1 on high-flow nasal cannula (HFNC). For those who developed PT, 4 were on NIV, 5 were on MV, and 1 was on NRFM. In patients who developed both PT and PM, 3 had different modes of ventilation when they developed each event (2 events on NIV, 2 events on MV, 2 events on HFNC), 8 had both events on the same mode with the same settings (6 NIV, 1 MV and 1 NRFM), 3 had them on the same mode with different settings (1 NIV, 2 MV), and 1 patient had missing data. Overall, out of 58 individual events, 44 developed in patients on positive pressure ventilation (NIV or MV), 12 developed on other modes of ventilation (HFNC or NRFM), and 2 had missing data.

## 4 DISCUSSION

Our study demonstrates that among patients with COVID-19 pneumonia, those who developed pneumothorax (PT), pneumomediastinum (PM), or both experienced significantly higher mortality. The development of PT or PM thus appears to reflect disease severity. Additionally, these events were associated with more extensive lung injury, as evidenced by the higher proportion of severe or critical involvement on chest CT in the group that developed events, as well as the increased risk of these events with higher CT severity scores. The scale we used was arbitrary; however, it is important to note that at the time of data collection and scan interpretation, no internationally validated scale existed for quantifying the severity of COVID-19 pneumonia. More recently, in 2024, Keskin *et al.* introduced the CT severity score (CTSS), which divides the lungs into 20 segments and grades the extent of involvement on a scale of 0–40 [9]. CTSS has shown a strong correlation with patient age, ICU admission, and need for ventilation, highlighting its prognostic value. Although this scoring system was not available to our radiologists at the time, the method we used to evaluate CT scans was comparable, and we believe the results would have been similar if CTSS had been applied.

Although several initial case reports and case series suggested a similar prognosis for patients with COVID-19 pneumonia who developed PT and PM compared to those without these events [10], more recent studies have reported findings similar to ours. In 2021, Marciniak et al analyzed the ISARIC WHO clinical characterization protocol in the United Kingdom. Their data showed a 0.97% incidence of PT in all COVID-19 patients and noted a worse prognosis in that group with an odds ratio of death of 2.94 [11]. Miro *et al.* reported that PT in COVID-19 as a direct presentation to the Emergency Department was a rare occurrence but was associated with a worse prognosis than COVID-19 patients without PT and non-COVID-19 patients with PT [12]. Woo *et al.* reviewed 17 case series and 87 case reports between January 2020 and December 2021 and found that PT with or without PM had a mortality of 23% [13]. Similarly, in a case-control study design, Bonato *et al.* found that patients with severe acute respiratory failure secondary to COVID-19 pneumonia who had PT and/or PM had higher in-hospital and 90-day mortality than matched controls [14]. Beletti *et al.* found a mortality of 60% in patients who developed PT or PM while on mechanical ventilation [15]. Finally, as mentioned previously, three systematic reviews regarding this topic found conflicting data on the prognostic implications of these events [6-8].

Most events in our population occurred on positive pressure ventilation (NIV or MV), which represents an expected finding. Our numbers show a similar incidence of events among patients on NIV and MV, differing from prior reports that showed the highest incidence to be on NIV [16].

It is important to mention that a significant number of events (12 of 56, 21.4%) occurred while patients were on other modes of ventilation (HFNC, NRFM, nasal oxygen). This important finding may guide physicians caring for patients with COVID-19 pneumonia, and highlights altered pulmonary mechanics even in patients not requiring positive pressure ventilation. Clinicians should maintain a high index of suspicion in cases of deterioration in these patients' respiratory status, regardless of the mode of ventilation, and a barotrauma event should be ruled out using chest imaging or bedside ultrasonography.

Overall, of the 43 patients who developed events, 15 were on mechanical ventilation at the time of occurrence. Outcomes for these patients were poor, and mortality was very high (14 deaths, 93%). For the ones not on MV at occurrence, 19 were on NIV (13 deaths - 68%), 2 were on HFNC (no deaths), 6 were on NRFM (2 deaths – 33%), and one patient had missing ventilation data. The finding of a significantly higher mortality when events occur on mechanical ventilation is in line with previous literature [17].

In the population of patients who developed either event, the fact that only three died within the first 48 hours following occurrence suggests that the events themselves did not cause mortality. Instead, they are strongly associated with death and likely constitute a marker of more severe underlying lung injury, which eventually leads to the demise of these patients. Events occurred more often with increasing age and with higher BMIs. This is in contrast to some reports where higher BMI had a protective effect [18]. The effect of obesity on critical illness has been the subject of debate for several years, with many reports suggesting an “obesity paradox” of better outcomes for obese patients with critical illness [19]. However, when COVID-19-specific hospitalizations were assessed in a large inpatient national sample study, obesity was associated with significantly worse outcomes [20]. The protective or detrimental effect of higher BMIs on barotrauma events in COVID-19 pneumonia will need to be confirmed in future trials.

Overall, our study contributes to the existing data on this important topic. Its cross-sectional design reflects real-life data. The main finding of a poor prognosis associated with these events was strongly significant. Such information may be helpful in triaging and closely monitoring these patients, as well as in discussions with patients and their families.

## 5 LIMITATIONS

The main limitation of our study is its retrospective nature. The cross-sectional design was employed to partially mitigate this issue, and the high number of patients and events encountered lends validity to the study and its results. Future prospective observational trials may further confirm these findings.

Finally, we acknowledge that our cohort had a high overall mortality at 28%. A possible explanation is that the data originate from a time when the lack of resources and available hospital beds throughout the country mandated that only the most severe cases be admitted to hospitals, while milder cases were treated at home, and widespread vaccination campaigns had not yet been implemented.

## CONCLUSION

Barotrauma events, particularly pneumothorax and pneumomediastinum, are well-known complications of COVID-19 pneumonia, occurring in 1% of mild infections and up to 15% of severe cases. Our cross-sectional analysis shows significantly higher mortality in patients who develop these events. Associated risk factors include older age, with a peak incidence in the sixth decade of life, and higher BMI. Patients do not typically die as a direct consequence of these events; rather, their occurrence serves as a marker of poor lung function and indicates a worse prognosis. A total of 79% of events develop during mechanical ventilation or non-invasive ventilation and are associated with very poor outcomes, while 21% occur with other modes of oxygenation and have a better overall prognosis. Future prospective trials are warranted to confirm these findings and further delineate prognostic indicators and associated factors.

## Figures and Tables

**Table 1:** Frequency of events in different comorbidities.

**Comorbidities**	**Patients with Event (%)**	***p*-value**
**Ever smoker**	7.1	0.42
**Non-smoker**	8.9	
**Diabetes mellitus**	8	0.93
**No diabetes mellitus**	8.2	
**Hypertension**	8	0.91
**No hypertension**	8.3	
**COPD**	10.5	0.70
**No COPD**	8.1	
**Chronic kidney disease**	15	0.50
**No chronic kidney disease**	7.9	
**Heart failure**	6.7	0.64
**No heart failure**	8.3	
**Coronary artery disease**	7.1	0.87
**No coronary artery disease**	8.4	
**Malignancy**	8.6	0.92
**No malignancy**	8.1	

## Data Availability

The data supporting the findings of the article will be available from the corresponding author [D.G] upon reasonable request.

## LIST OF ABBREVIATIONS

ARDS: Acute Respiratory Distress Syndrome
BMI: Body Mass Index
CAD: Coronary Artery Disease
CHF: Congestive Heart Failure
CKD: Chronic Kidney Disease
COPD: Chronic Obstructive Pulmonary Disease
COVID-19: Coronavirus 2019
CT: Computed Tomography
DM: Diabetes Mellitus
HF: Heart Failure
HFNC: High-Flow Nasal Cannula
HTN: Hypertension
MV: Mechanical Ventilation
NIV: Non-Invasive Ventilation
NRFM: Non-Rebreather Face Mask
PM: Pneumomediastinum
PT: Pneumothorax
RT-PCR: Reverse Transcription Polymerase Chain Reaction
SARS-CoV-2: Severe Acute Respiratory Syndrome Coronavirus 2
SPSS: Statistical Package for Social Sciences
WHO: World Health Organization

## STANDARDS OF REPORTING

STROBE guidelines were followed.
